# The Microalga *Skeletonema marinoi* Induces Apoptosis and DNA Damage in K562 Cell Line by Modulating NADPH Oxidase

**DOI:** 10.3390/molecules27238270

**Published:** 2022-11-27

**Authors:** Roberto Ciarcia, Consiglia Longobardi, Gianmarco Ferrara, Serena Montagnaro, Emanuela Andretta, Francesco Pagnini, Salvatore Florio, Lucianna Maruccio, Chiara Lauritano, Sara Damiano

**Affiliations:** 1 Department of Veterinary Medicine and Animal Productions, University of Naples “Federico II”, Via Delpino n.1, 80137 Naples, Italy; 2 Department of Mental, Physical Health and Preventive Medicine, University of Campania “Luigi Vanvitelli”, Largo Madonna delle Grazie n.1, 80138 Naples, Italy; 3 Department of Medicine and Surgery, Unit of Radiology, University of Parma, Via Università n. 12, 43126 Parma, Italy; 4 Ecosustainable Marine Biotechnology Department, Stazione Zoologica Anton Dohrn, Via Acton n. 55, 80133 Naples, Italy

**Keywords:** *Skeletonema marinoi*, leukemia, oxidative stress, apoptosis

## Abstract

Chronic myeloid leukemia (CML) is a myeloproliferative disease that activates multiple signaling pathways, causing cells to produce higher levels of reactive oxygen species (ROS). Nicotinamide adenine dinucleotide phosphate (NADPH) oxidases (NOXs) are a major generator of ROS in leukemia, and marine natural products have shown promising activities for the treatment of hematopoietic malignancies. In the present study, we investigated the effect of the marine microalga *Skeletonema marinoi* (S.M.), a ubiquitous diatom that forms massive blooms in the oceans, on the human leukemia cell line K562. The effects of S.M. extract on cell viability, production of ROS, nitric oxide (NO), and apoptosis were examined. In this preliminary work, S.M. was able to decrease cell viability (*p* < 0.05) and increase apoptosis levels (*p* < 0.05) in K562 cells after 48 h of treatment. In addition, the levels of NOX, NO, and malondialdehyde (MDA) were reduced in K562-treated cells (*p* < 0.05), whereas the levels of SOD, CAT, and GPx increased during treatment (*p* < 0.05). Finally, analyzing Bax and Bcl-2 expression, we found a significant increase in the proapoptotic protein Bax and a sustained decrease in the antiapoptotic protein Bcl-2 (*p* < 0.05) in the K562-treated cells.

## 1. Introduction

Cancer is the second leading cause of death in industrialized countries, after cardiovascular disease. Hematologic neoplasms are one of the most common causes of malignancies in children and young adults, with the highest mortality rate [1]. Chronic myeloid leukemia (CML) is a myeloproliferative disorder in which a reciprocal translocation between chromosomes 9 and 22 generates the chimeric kinase Bcr-Abl, which activates several signaling pathways, such as JAK/STAT, MEK/ERK, and PI3K/Akt [2]. These pathways’ activation leads cells to produce more reactive oxygen species (ROS) than untransformed cells [3].

Bcr-Abl is an important therapeutic target [1], and the development and clinical use of tyrosine kinase inhibitors (TKIs) have significantly improved the prognosis for CML patients [4]. Despite the important advances in prognosis, the currently available TKIs have several adverse effects, some of which can be attributed to the inhibition of off-target tyrosine kinases. Several tyrosine kinase inhibitors have been developed, but the problem of resistance and intolerance that develops over time is still a problem for patients with CML [1,4,5]. For this purpose, the discovery of new drugs with lower toxicity and higher efficacy is an important clinical task.

Studies suggest that excessive production of ROS induces oxidative stress, disrupts cellular homeostasis, and contributes to bone marrow failure and acute myeloid leukemia [5]. In biological systems, ROS is continuously produced and excreted. However, when the production of ROS is excessive, it causes oxidative stress, damages deoxyribonucleic acid (DNA), and contributes to the pathogenesis of various diseases, including cancer [6]. In hematological malignancies, ROS prevents apoptosis and promotes cell survival, growth, proliferation, migration, and drug resistance [6]. In CML, ectopic expression of Bcr-Abl has been shown to induce the production of ROS in hematopoietic cells [7], and the generation of ROS is thought to be critical for Bcr-Abl transformation [8]. Markers of oxidative stress, such as malondialdehyde (MDA) and protein-carbonyls, have been found in the plasma of CML patients compared with healthy ones, and the extent of oxidative stress has shown a rise during the accelerated phase of CML [9,10,11,12]. Moreover, nitric oxide (NO), a multifunctional transcellular messenger involved in numerous physiological and pathological conditions, has shown to be implicated in the apoptosis process in human cell lines from patients diagnosed with leukemia and lymphoma [13].

Nicotinamide adenine dinucleotide phosphate (NADPH) oxidases (NOX) are an important source of ROS in leukemia [14]. Leukemic blasts continuously generate ROS, which are involved in the regulating intracellular signaling pathways and modulating cells in the microenvironment, thereby promoting leukemogenesis [14]. The NOX family, which consists of seven members, is responsible for the increase in ROS production after Bcr-Abl induction, but the exact mechanism of action is still unclear [14]. The NOX complex is activated when the catalytic subunit (gp91-phox or NOX2) is bound to the p22-phox proteins, migrates to the plasma membrane, and binds the cytosolic subunits (p47-phox, p67-phox, p40-phox, and the GTPase Rac) [14]. Moreover, several experimental approaches demonstrated that the production of ROS is a consequence of a p22-phox-dependent NADPH oxidase [15].

Marine natural products (MNPs) have proven to be promising agent for the treatment of hematopoietic malignancies [16]. The first drug of marine origin, Cytarabine (Ara-C), was originally isolated from the sponge *Cryptotethya crypta* and approved by the Food and Drug Administration (FDA) in 1969 for the treatment of acute myelocytic leukemia and non-Hodgkin’s lymphoma [17]. Recently, Polatuzumab vedotin (DCDS-4501A) was approved by the FDA in 2019 for the treatment of non-Hodgkin’s lymphoma, chronic lymphocytic leukemia, lymphoma, and B-cell lymphoma [18], and Belantamab mafodotin was approved by FDA in 2020 for the treatment relapsed/refractory multiple myeloma [19]. In recent years, the discovery of marine-derived drugs also focused on microorganisms, as they are considered eco-friendly and eco-sustainable potential sources of new compounds for cancer treatment. Compared to classical macroorganisms (such as sponges), they are easier to cultivate in closed photobioreactor systems or open ponds but still poorly studied for drug discovery. In this preliminary work, we investigated the effects of extracts of the marine diatom *Skeletonema marinoi* (S.M.) on the human immortalized myeloid leukemia cell line K562. S.M. is a ubiquitous diatom that forms massive blooms in many coastal seas of the world [20]. Previous studies have focused on the genetic structure of this diatom [21], its responses to environmental stressors [22,23], interactions with predators [24], production of secondary metabolites [25], and potential bioactivities for the treatment of human diseases such as cancer, microbial infections, and neurodegenerative disorders [26,27,28,29]. Miralto and coworkers [20] isolated three polyunsaturated aldehydes (PUAs), namely 2*E*,4*Z*,7*Z*-decatrienal, 2*E*,4*E*,7*Z*-decatrienal, and 2*E*,4*E*-decadienal, from the marine diatoms *Thalassiosira rotula*, *Skeletonema costatum*, and *Pseudo-nitzschia delicatissima*. PUAs belong to a class of compounds called oxylipins, which are derived from the oxidation of polyunsaturated fatty acids. After this discovery, several other oxylipins were identified [25,30,31]. It has been shown that the production of oxylipins occurs when algal cells are damaged by grazing predators, such as copepods, or under stressful conditions (e.g., at the end of stationary phase, when nutrients decrease inducing cell death), suggesting that their activity in the sea is mostly defensive against predators. Indeed, studies have shown that oxylipins reduce egg production by predators, decrease hatching success, trigger the birth of malformed nauplii, and alter the expression of stress-responsive genes in the predator [20,24,32], indicating its role in shaping plant–animal interactions in the oceans.

In addition to oxylipins, S.M. has been demonstrated a promising source of other bioactive compounds, such as glycerolipids, sterol sulphates, fatty acids, ascorbic acid, and phenolic compounds that may have antioxidant and antiproliferative properties [28,29]. Regarding antiproliferative activity, S.M. extracts have previously exhibited bioactivity against human melanoma cells A2058 when cultured at low temperature and high light [22] or under nitrogen-starvation conditions [23]. Moreover, their selective cytotoxic activity against the hematologic cancer U-937 cells and the colorectal cancer HCT-116 cell line was demonstrated by Miceli et al. [26]. In the present pilot study, we investigated, for the first time, the effects of S.M. extracts on cell viability, ROS production, and apoptosis in CML cells K562. Specifically, we investigated the relationship between ROS production and apoptosis through the activity of NOX, which provided insight into a novel therapeutic mechanism for the treatment of CML.

## 2. Results

### 2.1. The Extract of Skeletonema marinoi Affects the Viability of K562 Cells

**Figure 1:**
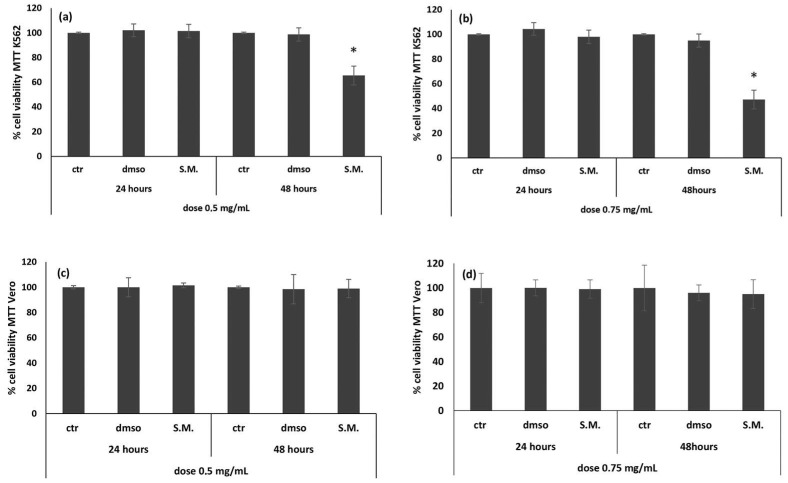
Effect of *Skeletonema marinoi* (S.M.) on Vero (**c**,**d**) and K562 (**a**,**b**) cells viability. MTT test was performed at 24 h and 48 h, using two different concentrations of S.M. (0.5 and 0.75 mg/mL). Data are expressed as a percentage of the cell viability, and results are expressed as the mean ± standard deviation (SD) of three experiments. Statistical differences between control and S.M. were evaluated by one-way analysis of variance (ANOVA), followed by Tukey’s posttest (* *p* < 0.05 versus ctr/dmso cells).

The MTT (3-(4,5-dimethylthiazol-2-yl)-2,5-diphenyltetrazolium bromide) assay was performed on K562 and Vero cells after 24 and 48 h of treatment with two different dosages of S.M. extract: 0.5 and 0.75 mg/mL. Treatment of K562 cells resulted in a time- and dose-dependent viability decrease. In particular, a significant reduction of cell viability after 48 h of incubation with both concentrations was observed (Figure 1a,b). Since the best result was obtained at 48 h with a concentration of 0.75 mg/mL (Figure 1b), this combination was used for the subsequent experiments. There were no significant differences between ctr/dmso and treated Vero cells (Figure 1c,d).

### 2.2. The Extract of Skeletonema marinoi Induces Apoptosis in K562 Cells

**Figure 2:**
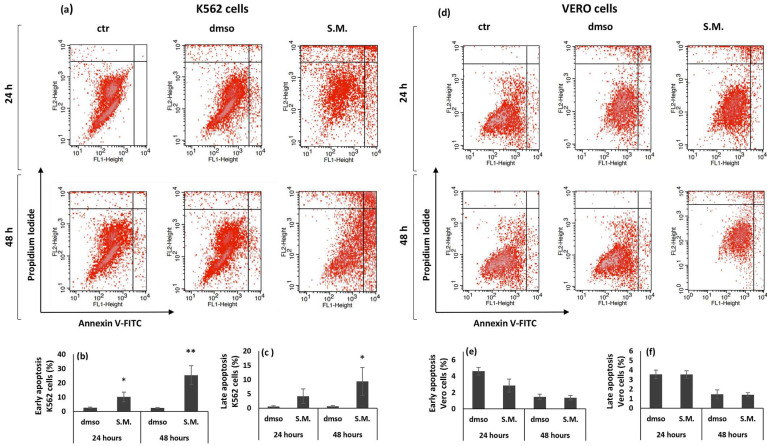
Cytofluorimetric measurement of apoptosis induced by *Skeletonema marinoi* (S.M.) (0.75 mg/mL) on K562 and Vero cells. Panel (**a**,**d**): scatter plots of the annexin V-FITC/PI flow cytometry graphs by FACS. The lower right quadrants represent cells in the early stage of apoptosis. The upper right quadrants represent cells in the late stage of apoptosis. Representative quantification of early (**b**,**e**) and late (**c**,**f**) apoptosis in dmso- and S.M.-treated K562 and Vero cells. Data are expressed as the mean ± standard deviation (SD) of three experiments. Statistical differences between control and S.M. were evaluated by Student’s *t*-test (* *p* < 0.05 versus dmso; ** *p* < 0.01 versus dmso).

Annexin-V/ PI (propidium iodide) staining quantitatively measured the apoptotic effect of S.M. extract (0.75 mg/mL) on K562 and Vero cells. As shown in Figure 2b, the number of early apoptotic K562 cells significantly increased both 24 and 48 h post treatment. Late apoptotic K562 cells significantly increased with incubation time and became significant at 48 h post S.M. treatment compared to dmso-treated cells (Figure 2c). The percentage of early and late apoptotic cells was not significantly increased in Vero treated with S.M. compared to the dmso group (Figure 2d–f).

### 2.3. The Extract of Skeletonema marinoi Protects K562 Cells from Lipid Peroxidation

**Figure 3:**
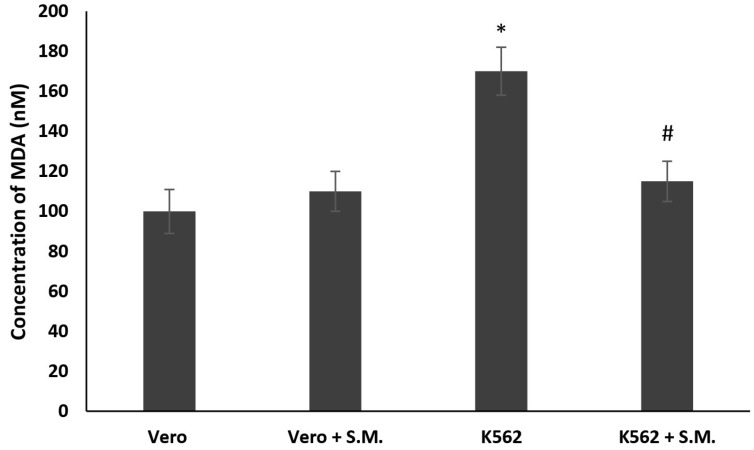
Lipid peroxidation inhibitory activity in K562 cells and Vero cells treated with *Skeletonema marinoi* (S.M.) for 48 h at a concentration of 0.75 mg/mL. Data are expressed by the means ± standard deviation (SD) of three experiments. (* *p* < 0.05 means a significant difference between the Vero and K562 cells; ^#^ *p* < 0.05 means a significant difference between the treated and untreated K562 cells).

MDA (malondialdehyde) production increased in K562 cells compared with Vero after 48 h (*p* < 0.05), showing a clear involvement of the pathway of the ROS (reactive oxygen species). The treatment of K562 with 0.75 mg/mL of S.M. extract demonstrates a protective effect against lipid peroxidation induced by H_2_O_2_ after 48 h (*p* < 0.05) compared with the untreated ones (Figure 3). No significant effect was observed between Vero cells and S.M.-treated Vero cells.

### 2.4. The Extract of Skeletonema marinoi Exerts a Protective Effect on Nitrites (NO2^−^) and Nitrates (NO3^−^) Production

**Figure 4:**
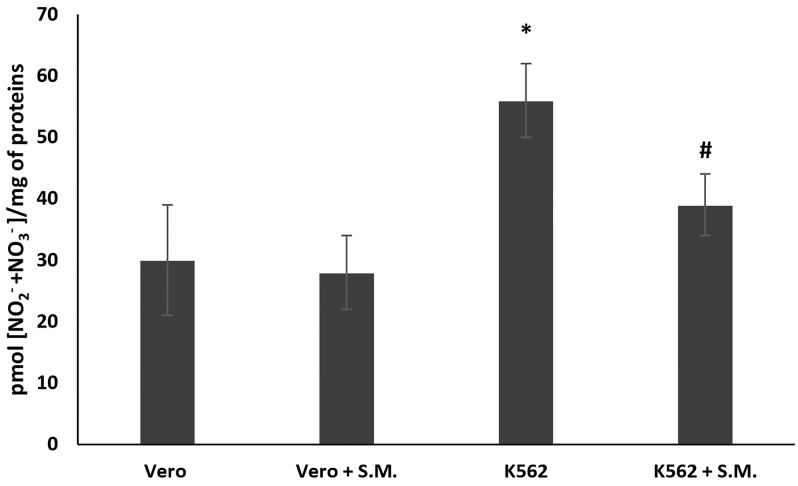
Effect of *Skeletonema marinoi* (S.M.) on nitric oxide (NO) production in K562 and Vero cells. After 48 h of treatment with 0.75 mg/mL of S.M., nitrites (NO_2_^−^) and nitrates (NO_3_^−^) levels were assayed by the Griess reagent. Data were represented by the means ± standard deviation (SD) of three experiments. (* *p* < 0.05 means a significant difference between the Vero and K562 cells; ^#^ *p* < 0.05 means a significant difference between the treated and untreated K562 cells).

In K562 cells, the NO_2_^−^ and NO_3_^−^ levels increased compared to Vero cells after 48 h (Figure 4, *p* < 0.05), demonstrating the involvement of NO production in hematological cancer. Moreover, as shown in Figure 4, S.M. extract (0.75 mg/mL) exhibited a protective effect against NO productions (Figure 4, *p* < 0.05 compared with untreated K562 cells) after 48 h of incubation, but no significant effect was observed in S.M.-treated Vero cells.

### 2.5. The Redox Status Activity Is Restored in K562 Cells after Treatment with the Extract of Skeletonema marinoi

**Figure 5:**
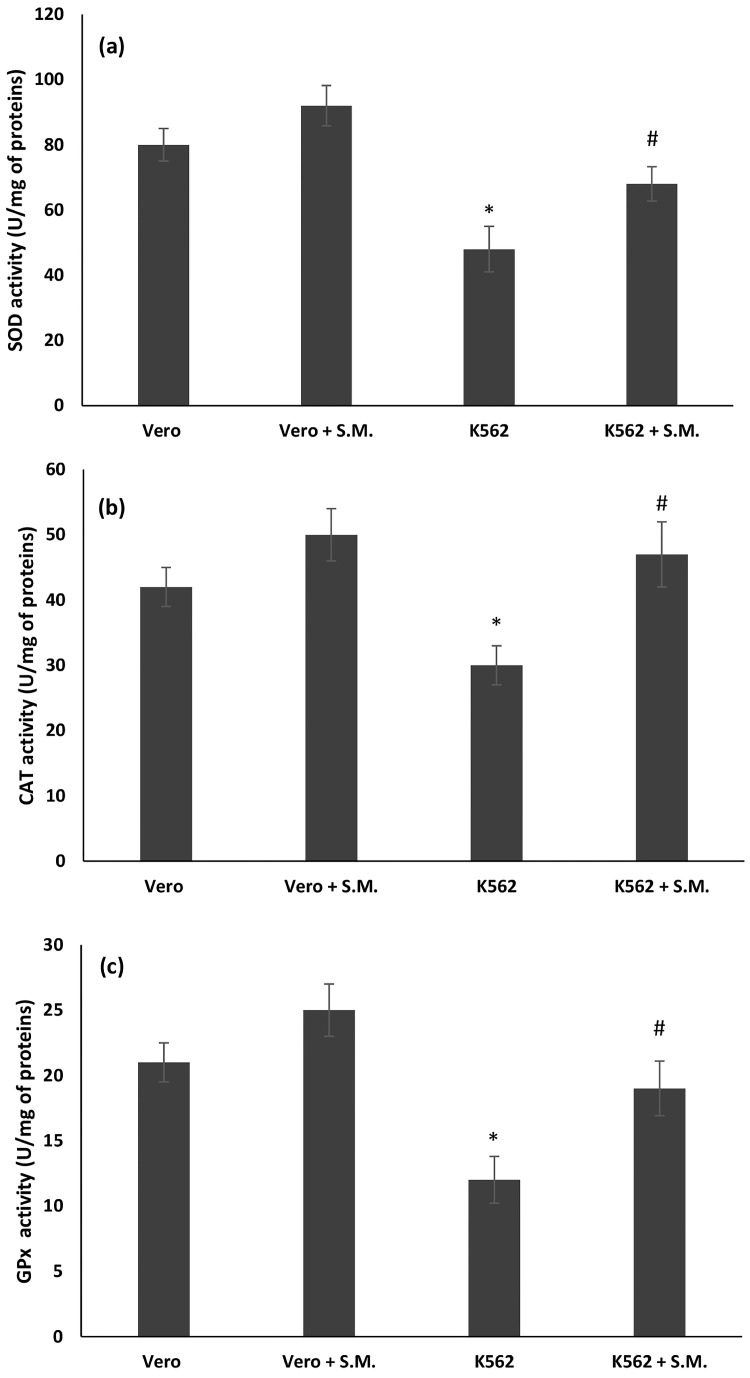
Effects of *Skeletonema marinoi* (S.M.) on (**a**) superoxide dismutase (SOD), (**b**) catalase (CAT), and (**c**) glutathione peroxidase (GPx) activities expressed as units for milligrams mg of proteins (U/mg of proteins) in Vero and K562 cells after 48 h incubation with S.M. extract (0.75 mg/mL). Data were represented by the means ± standard deviation (SD) of three experiments (* *p* < 0.05 means a significant difference between the Vero and K562 cells; ^#^ *p* < 0.05 means a significant difference between the treated and untreated K562 cells).

The antioxidant markers superoxide dismutase (SOD), catalase (CAT), and glutathione peroxidase (GPx) in the treated and untreated Vero and K562 cells are shown in Figure 4. The activities of SOD, CAT, and GPx were significantly decreased in the untreated K562 cells compared with the Vero cells (Figure 5, *p* < 0.05). Treatment with the microalga resulted in a significant recovery of SOD, CAT, and GPx levels compared to the untreated K562 cells (*p* < 0.05). In the Vero cells, S.M. showed no differences between the Vero treated and untreated cells (Figure 5).

### 2.6. Oxidative DNA Damage Is Decreased in K562 Cells after Treatment with the Extract of Skeletonema marinoi

**Figure 6:**
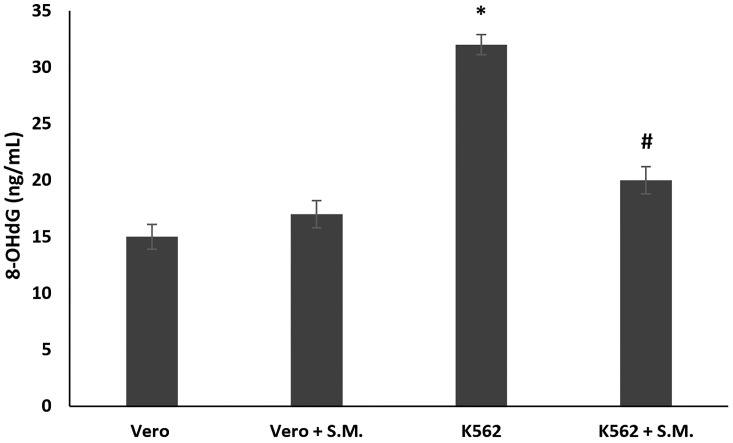
Effects of *Skeletonema marinoi* (S.M.) (0.75 mg/mL) on oxidative DNA damage of K562 and Vero cells after 48 h of treatment. Data were represented by the means ± standard deviation (SD) of three experiments. (* *p* < 0.05 means a significant difference between the Vero and K562 cells; ^#^ *p* < 0.05 means a significant difference between the treated and untreated K562 cells).

The 8-OHdG (8-hydroxy-2′-deoxyguanosine), a marker for oxidative DNA damage, was measured in treated and untreated K562 cells. 8-OHdG levels were significantly higher in K562 untreated cells with respect to Vero cells used as control. S.M. treatment (0.75 mg/mL, 48 h) significantly reduced 8-OHdG levels when compared with the K562 untreated cells (Figure 6, *p* < 0.05).

### 2.7. The Extract of Skeletonema marinoi Induces K562 Cell Apoptosis and Reduces Oxidative Stress through NOX2 Pathway

**Figure 7:**
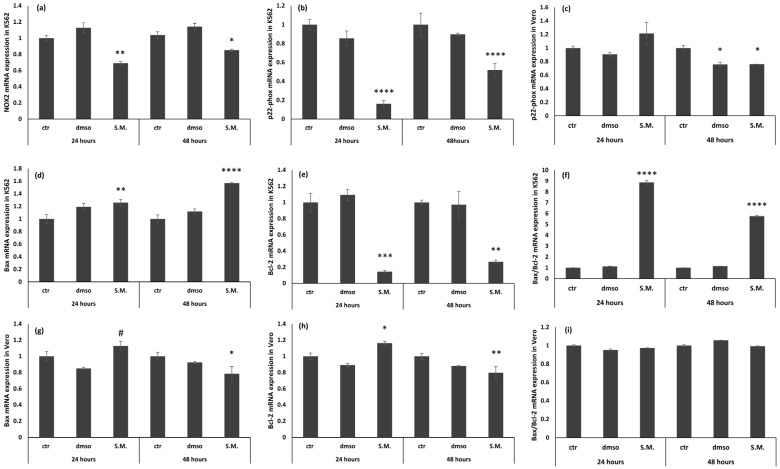
Effects of *Skeletonema marinoi* (S.M.) extract (0.75 mg/mL) on NOX2, p22-phox, Bax, Bcl-2, and Bax/Bcl-2 ratio mRNA expression in K562 cells and Vero cells. (**a**) NOX2 mRNA expression in K562; (**b**) p22-phox mRNA expression in K562; (**c**) p22-phox mRNA expression in Vero; (**d**) Bax mRNA expression in K562; (**e**) Bcl-2 mRNA expression in K562; (**f**) Bax/Bcl-2 ratio mRNA expression in K562; (**g**) Bax mRNA expression in Vero; (**h**) Bcl-2 mRNA expression in Vero; and (**i**) Bax/Bcl-2 ratio mRNA expression in Vero. Data are expressed as mean ± standard deviation (SD) of three experiments and compared with *t*-test (* *p* < 0.05 versus control cells; ** *p* < 0.01 versus control cells; *** *p* < 0.001 versus control cells; **** *p* < 0.001 versus control cells; ^#^ *p* < 0.05 versus dmso treated cells).

The mRNA levels of NOX2, p22-phox, Bax, Bcl-2, and GAPDH genes in K562 and Vero cells were analyzed by real-time quantitative PCR. The data showed that the levels of NOX2, p22-phox, and Bcl-2 were increased in K562 cells compared with K562 cells treated with 0.75 mg/mL of S.M. (Figure 7, *p* < 0.05 compared with untreated K562 cells), demonstrating the protective effect of the extract on the formation of ROS and the induction of apoptosis. In addition, Bax levels increased in K562-treated cells, showing the key role of apoptosis in the development of chronic myeloid leukemia, and the results of this work demonstrate the protective effect of S.M. in the process of apoptosis (Figure 7, *p* < 0.05 vs. untreated K562 cells). Finally, the levels of NOX2 gene, p22-phox, Bax, and Bcl-2 were also examined in Vero cells, and no differences were found between treated and un-treated ones, showing that S.M. does not exert toxic effects on normal cells at the dose used in these experiments (Figure 7).

### 2.8. The Extract of Skeletonema marinoi Affects NOX2, p22-phox, and Apoptosis Proteins’ Expression in K562 Cells

**Figure 8:**
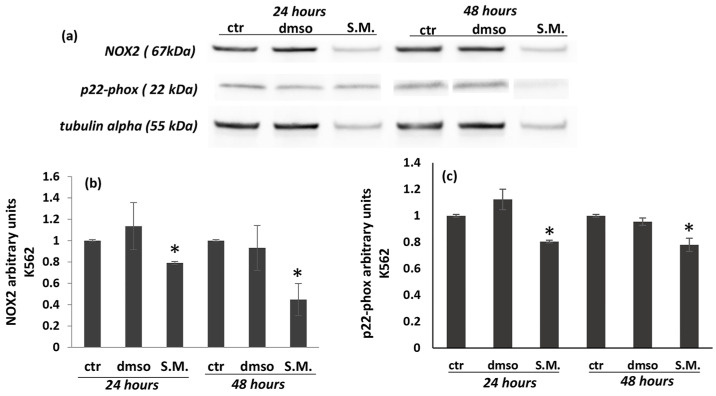
Effect of *Skeletonema marinoi* (S.M.) on NOX2 and p22-phox protein levels in K562 cells after 24 and 48 h of treatment. Panel (**a**): representative Western blot of NOX2 and p22-phox in K562 cells; panel (**b**): densitometric analysis of NOX2; panel (**c**): densitometric analysis of p22-phox. Experiments were conducted in triplicates, and the values were normalized towards tubulin. Densitometric analyses were expressed as arbitrary units. Data are shown as mean ± standard deviation (SD) and were compared by ANOVA (* *p* < 0.05 versus untreated K562 cells).

**Figure 9:**
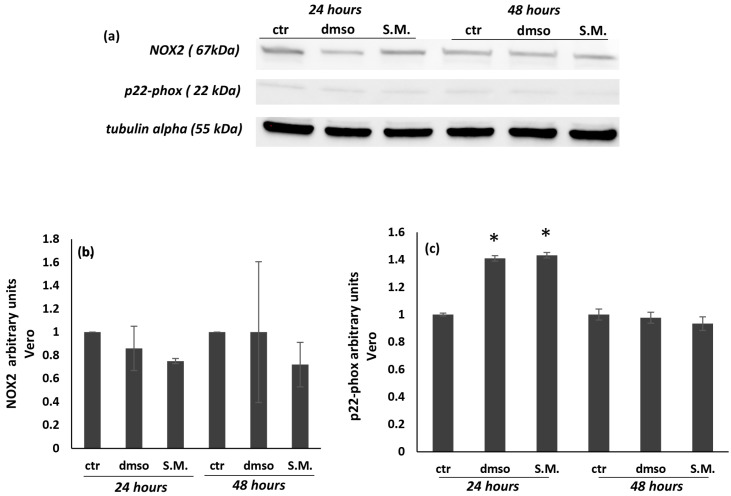
Effect of *Skeletonema marinoi* (S.M.) on NOX2 and p22-phox protein levels in Vero cells after 24 and 48 h of treatment. Panel (**a**): representative Western blot of NOX 2 and p22-phox in Vero cells; panel (**b**): densitometric analysis of NOX2; panel (**c**): densitometric analysis of p22-phox. Experiments were conducted in triplicates, and the values were normalized towards the tubulin. Densitometric analyses were expressed as arbitrary units. Data are shown as mean ± standard deviation (SD) and were compared by ANOVA (* *p* < 0.05 versus untreated Vero cells).

In this work, the levels of NOX2 and its catalytic subunit p22-phox were analyzed by Western blot, showing that S.M. (0.75 mg/mL) was able to reduce both protein levels in K562 cells (Figure 8a–c), while no significant effects were shown in Vero cells (Figure 9a–c). Taken together, these results suggest the existence of interactions between the regulation of ROS production and the apoptotic pathway.

**Figure 10:**
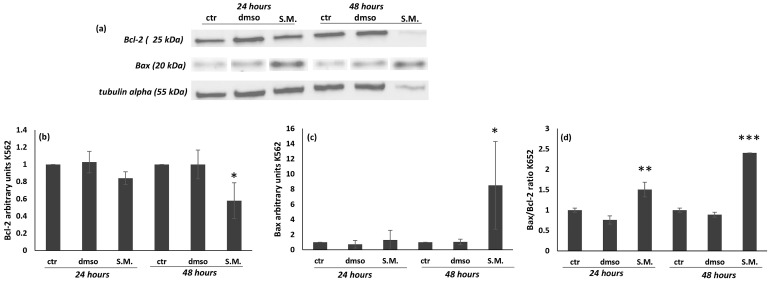
Effect of *Skeletonema marinoi* (S.M.) (0.75 mg/mL) on Bax and Bcl-2 protein levels in K562 cells after 24 and 48 h of treatment. Panel (**a**): representative Western blot of Bax and Bcl-2; panel (**b**): densitometric analysis of Bax; panel (**c**): densitometric analysis of Bcl-2; and panel (**d**): densitometric analysis of the Bax/Bcl-2 ratio. Experiments were conducted in triplicates, and the values were normalized towards the tubuline. Densitometric analyses were expressed as arbitrary units. Data are shown as mean ± standard deviation (SD) and were compared by ANOVA (* *p* < 0.05 versus untreated Vero cells; ** *p* < 0.01 versus untreated Vero cells; *** *p* < 0.001 versus untreated Vero cells).

**Figure 11:**
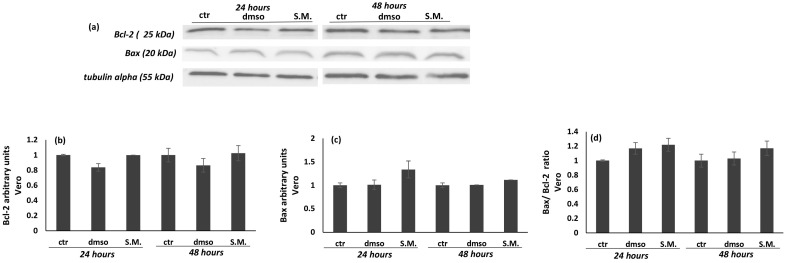
Effect of *Skeletonema marinoi* (S.M.) (0.75 mg/mL) on Bax and Bcl-2 protein levels in Vero cells after 24 and 48 h of treatment. Panel (**a**): representative Western blot of Bax and Bcl-2; panel (**b**): densitometric analysis of Bax; panel (**c**): densitometric analysis of Bcl-2; and panel (**d**): densitometric analysis of the Bax/Bcl-2 ratio. Experiments were conducted in triplicates, and the values were normalized towards the tubuline. Densitometric analyses were expressed as arbitrary units. Data are shown as mean ± standard deviation (SD) and were compared by ANOVA.

Figure 10 showed that the levels of Bax after treatment with S.M. (0.75 mg/mL) on K562 cells induced a significant increase in the expression; moreover, a suppression of Bcl-2 was observed after 48 h of treatment (Figure 10a–c). In contrast, no effect was observed in Vero cells treated with S.M. (Figure 11a–c). It is interesting to note that the ratio Bax/Bcl-2 is also significatively increased in K562-treated cells in respect to untreated ones (Figure 10d), and no effect was observed in the Bax/Bcl-2 ratio of Vero cells treated with same concentration of S.M. (Figure 11d).

## 3. Discussion

Microalgae are heterogeneous microorganisms that are an excellent source of carotenoids, polysaccharides, vitamins, and other fine chemicals for nutraceutical and cosmetic applications [27]. Among them, S.M. [22] has shown bioactivity against human melanoma cell lines (A2058) and selective cytotoxic activity against hematological cancer cell line (U-937) and colon cancer cells (HCT-116) compared to healthy human mesenchymal ones (MePR-2B) [26]. In this study, we described for the first time the possible mechanism of action of S.M. in the human chronic myeloid leukemia K562 cell line.

The results showed that S.M. inhibited the growth of K562 cells and that the inhibition of cell growth was strictly in accordance with the exposure time and tested concentrations (Figure 1, *p* < 0.05). In addition, our study also showed that S.M. extract had no cytotoxic effects on normal Vero cells. Induction of apoptosis is a possible mechanism by which the antiproliferative activity of S.M. might be exerted in K562 cells. To this end, we investigated the apoptotic signaling pathways involved. Studies in the literature have shown that Bcl-2 family proteins induce the activation of the intrinsic apoptotic pathway mediated by mitochondria as one of the main elements for anticancer drug function [33,34]. Here, we investigated the effects of algal extract on the expression of the proapoptotic protein Bax and the antiapoptotic protein Bcl-2 and found a significant increase in Bax expression (Figure 10, *p* < 0.05), while Bcl-2 expression was inhibited (Figure 1, *p* < 0.05). Therefore, the Bax/Bcl-2 ratio was also increased, and these data play a key role in the activation of the mitochondrial apoptotic pathway (Figure 10, *p* < 0.001). Indeed, Bax/Bcl-2 regulates the release of Cyt C into the cytosol, leading to cell apoptosis [34]. There are numerous data in the literature supporting the close relationship between Bax and Bcl-2. Several biological systems indicate that cells are protected when there is an excess of Bcl-2, whereas cells are prone to apoptosis when there is an excess of Bax [35]. Moreover, studies of Bcl-2-deficient mice at one week of age show growth retardation and develop renal hypoplasia, polycystic kidneys, fulminant apoptosis of the thymus and spleen, and lymphocyte loss, demonstrating that alteration of the Bax/Bcl-2 ratio promotes cell death [36]. 

Several research groups have shown that NO interferes with the action of anticancer drugs [37,38], and its overproduction can result in apoptosis and DNA or mitochondrial membrane damage [39]. NO toxicity could be dependent on the NO and superoxide radical reaction to generate the pro-oxidant peroxynitrite, which is rapidly decomposed to the nitro radical, giving rise to nitrosative stress and DNA damage [40]. In particular, the DNA injury could be promoted by ROS and RNS, developing 8-OHdG and oxidative and nitrosative markers stress, respectively [41]. In the present work, we found an increase in 8-OHdG expression in untreated K562 cells with respect to Vero cells, and the S.M. treatment significantly reduced this 8-OHdG over-expression, exhibiting the protective action of the extract against the DNA damage in K562 cancer cells, suggesting potential use as an anticancer drug alone or in combination with other cytotoxic agents (Figure 5, *p* < 0.001).

Does ROS play a key role in CML? In many pathological conditions, including cancer, excessive ROS production related to a lack of antioxidant defenses has been well-demonstrated [42]. Although the relationship between oxidative stress and malignancy has not been clearly established, several lines of evidence suggest that ROS may promote cell survival, proliferation, and drug resistance [43,44]. In CML, oxidative stress increases during the accelerated phase of CML [37], and ectopic expression of Bcl-Abl alone has been able to induce the production of ROS in hematopoietic cells [45]. In addition, when compared to control subjects, the levels of MDA and prostone carbonys, which are markers of oxidative stress, were increased in the plasma of patients with CML [46,47]. In the current preliminary study, we observed a sharp increase in MDA levels in untreated K562 cells (Figure 3, *p* < 0.05), which is consistent with data in the literature. Interestingly, it was also observed that the S.M. extract used in this work significantly reduced MDA levels (Figure 3, *p* < 0.05).

In general, cells have a sophisticated antioxidant defense system consisting of enzymatic ROS scavengers, such as GPx, SOD, and CAT. Accordingly, the effect of S.M. on antioxidant defense activity was investigated. A significant decrease in GPx, SOD, and CAT activity was observed only in the untreated K562 cells (Figure 3, *p* < 0.05). Antioxidant defense system levels were restored during S.M. treatment in K562 cells, but no effects were observed in Vero-treated cells. Antioxidants can curb the negative effects of ROS activity, such as the degenerative incidence of cancer. However, if the action of free radicals is prolonged, the capacity of the defense system against ROS may be overwhelmed, leading to the onset of the disease [45]. Under physiological conditions, the moderate balance between pro-oxidant and antioxidant compounds favors the pro-oxidant ones, generating a mild oxidative stress that triggers the involvement of the endogenous antioxidant systems of the organism [45]. In this work, we observed that S.M. acts as an exogenous antioxidant and favors the restoration of pro-oxidant factors.

The sources of ROS production in CML patients remain unclear, but increased NOX activity and ROS production have been reported [47]. Several data have shown that the main sources of ROS are mitochondria, and the NOX family has emerged as a major player [13,14]. It has been shown that NOX isoenzymes increase in association with ROS production and tumor activity in various cancer cells [13,14]. In this work, NOX2 activity measured by Western blot was increased in untreated K562 cells with respect to control Vero cells (Figure 7 and Figure 8, *p* < 0.05). In contrast, a significant decrease in NOX2 expression was observed when cells were treated with S.M. compared to untreated cells. Moreover, the reduction of ROS is related to the downregulation of p22-phox in K562 cells during S.M. treatment. Taken together, these results describe for the first time a possible anti-apoptotic and anti-oxidative effect of S.M. against CML and the possibility to use this extract for further studies to identify potential therapeutic agents in CML patients.

According to Persistence Market Research (https://www.persistencemarketresearch.com/market-research/microalgae-market.asp; accessed on 24 June 2022), the global market for microalgae-based products is expected to reach USD 5 billion during the projected period 2021–2031 (5.7% compound annual growth rate or CAGR). Currently, there are no microalgae-based drugs on the market, but there are several microalgae-based products in the dietary supplement, cosmetics, and aquaculture sectors, especially thanks to their antioxidant and anti-inflammatory effects. The road to the pharmaceutical market is still long, but considering the strong activities of microalgae extracts, fractions, and compounds for various human diseases, we expect that there will soon be new microalgae extracts-based drugs. In addition, the Food and Drug Administration (FDA) has designated a number of microalgae species (biomass or extracts) as GRAS (generally recognized as safe), which means they are “safe to consume” [48]. Indeed, products based on microalgae-derived powders are now commercially available, and more studies and trials are underway for functional foods such as fresh pasta, cookies, and yogurt [49,50]. All these efforts and interesting bioactivities point to a growing market for microalgae in the near future.

## 4. Materials and Methods

### 4.1. Cell Lines and Reagents

The diatom *Skeletonema marinoi* (S.M.) used in this study is the CCMP2092 strain (NCMA at Bigelow Laboratory, East Boothbay, ME, USA). The human cell line K562 and mammalian Vero cells (African green monkey kidney cells) were purchased from the Department of Veterinary Medicine and Animal productions (Naples, Italy). Chemical reagents were purchased from Sigma-Aldrich (Milan, Italy). Annexin V-FITC was purchased from Cell Signaling Technology (Beverly, MA, USA). Antibodies were purchased from Cell Signaling Technology (Beverly, MA, USA) or Santa Cruz Biotechnology (Heidelberg, Germany). 

### 4.2. Microalga Culturing

S.M. was cultured in Guillard’s F/2 medium, and experimental culturing was performed in triplicate in 10-L polycarbonate bottles, with constant bubbling with air filtered through 0.2 μm membrane filters in a climate chamber at 19 °C on a 12:12 h light: dark cycle and at 100 μmol photons m^−2^ s^−1^. For each bottle, initial cell concentration was 5000 cells/mL. Each day, 2 mL of microalgal culture was fixed with about 40 µL Lugol (final concentration of about 2%) and counted in a Bürker counting chamber under an Axioskop 2 microscope (20×) (Carl Zeiss GmbH, Oberkochen, Germany). At the end of the stationary phase cultures were centrifuged for 15 min at 4 °C at 1900× *g* (Eppendorf, 5810R, Hamburg, Germany). The stationary phase was selected because it is known to contain the highest production of secondary metabolites. Supernatants were discarded, and pellets were immediately frozen in liquid nitrogen and stored at −80 °C until use.

### 4.3. Algal Pellet Extraction

Microalgal pellets were extracted as in Martinez et al. [51]. Briefly, wet pellets (80% water content) were extracted by soaking in methanol (proportion 1:5, *w*/*v*) and 30 min of maceration. Samples were vortexed for 1 min, sonicated for 30 s, and centrifuged at 3000 rpm at 4 °C. The pellet obtained was discarded, while the organic phase was transferred into a spherical Pyrex flask and dried using a rotary evaporator.

### 4.4. Cell Culture

The erythroleukemia cell line (K562, ATCC Cat# 300224/p473ATCC RRID:CVCL_0004), was cultured (humidified atmosphere at 37 °C in 5% CO_2_) in 75 cm^2^ flasks in the presence of Iscove Modified Dulbecco Media (IMDM, Corning) supplemented with 10% fetal calf serum (Corning) and 100 U/mL streptomycin/penicillin.

African green monkey kidney cells (Vero, ATCC Cat# CCL-81, RRID:CVCL_0059) were used as the control cell line. Vero cells were cultured in Dulbecco′s Modified Eagle′s Medium (DMEM, Corning) supplemented with fetal calf serum and antibiotics, as previously described for IMDM.

When the cells reached a confluency of 3 × 10^5^/mL cells, a solution of alga extract solubilized in dimethyl sulfoxide (dmso) at a concentration of 0.5 mg/mL and 0.75 mg/mL was supplemented to the flasks and incubated for 24 and 48 h.

### 4.5. 3-(4,5-dimethylthiazol-2-yl)-2,5-diphenyltetrazolium Bromide (MTT) Assay

K562 and Vero cells were seeded in 96-well plates in a volume of 100 µL and a confluency of 4 × 10^5^/mL. A solution of S.M. extract (0.5 and 0.75 mg/mL) solubilized in dimethyl sulfoxide (dmso) was supplemented to the wells and incubated for 24 and 48 h. The cytotoxicity of S.M. extract was assessed using the MTT (SERVA) assay, as described in a previous work [52]. Briefly, after the incubation, 100 µL of MTT reagent (0.5 mg/mL in IMDM) was added to each well, and the plate was incubated for 4 h at 37 °C. The MTT was converted to purple formazan by mitochondrial dehydrogenase, and the formed formazan crystals were solubilized by adding 100 µL of dmso (Serva). Absorbance intensity was measured at 570 nm using Glomax Multi Detection System (Promega Corporation, Milan, Italy). Values were expressed as a percentage of the cell proliferation compared to control cells treated with dmso.

### 4.6. Annexin V-FITC Apoptosis Analysis

After 24 and 48 h of S.M. extract (0.75 mg/mL) treatment, a cytofluorimetric analysis of K562 and Vero cells (ctr, dmso, and S.M.) was performed to determine the percentage of apoptotic cells. A Cell Signaling Annexin V-FITC kit (Annexin V-FITC Early Apoptosis Detection Kit #6592) was used as described by the manufacturer. Briefly, after the incubation, cells were collected by centrifugation (500× *g* for 5 min), washed with ice-cold PBS 1X, and resuspended in Annexin binding buffer at 10^5^ cells/mL. Then, 12.5 µL PI and 1 µL Annexin V-FITC conjugate were added to 96 µL of cell solution. After 10 min on ice in the dark, the Annexin binding buffer was filled to a final volume of 250 µL, and each sample was analyzed immediately by FACS (BD FACSCalibur, BD Bio-sciences). Cells stained with both PI and Annexin V-FITC resulted in late apoptosis/necrotic stage, while cells stained with Annexin V-FITC were in early apoptotic stage. A total of 8000 events were recovered, and the data were analyzed with the BD cellQuest Pro software version 3.3 (BD Biosciences, San Jose, CA, USA).

### 4.7. Determination of Lipid Peroxidation

Lipid peroxidation was determined according to Ohkawa et al. [53]. After treatment with S.M. extract (0.75 mg/mL) for 48 h, K562 and Vero cells were incubated with 50 μM H_2_O_2_ for 2 h, washed with PBS, pelleted, and homogenized in 1.15% KCl. Then, samples were incubated with 0.2 mL of 8.1% SDS, 1.5 mL of 20% acetic acid, and 1.5 mL of 0.8% thiobarbituric acid with a final volume of 4 mL with distilled water and heated to 95 °C for 120 min. Then, 5.0 mL of *n*-butanol and pyridine (15:1 *v*/*v*) was added to each sample. Finally, samples were centrifugated at 825× *g* for 10 min, and the supernatant was isolated. The absorbance was measured at 532 nm. The lipid peroxidation effect was expressed as equivalent of MDA. Data were reported as mean ± SD.

### 4.8. Determination of NO_2_^−^ and NO_3_^−^ Productions

The supernatant of K562 cells and Vero cells, untreated and treated for 48 h with S.M. extract (0.75 mg/mL), was incubated with Griess’s reagent (1 part 0.75% sulfanilamide in 0.5 N H_3_PO_4_ and 1 part N-(1-naphthyl)-ethylenediamine dihydrochloride 0.75% in water) at 25 °C under reduced light for 20 min to determine the production levels of NO_2_^−^ and NO_3_^−^ [54]. The absorbance was read at 550 nm using Glomax Multi Detection System Spectrophotofluorometer (Promega Corporation, Milan, Italy), and the concentration of the analytes was calculated using a calibration curve (range 0.125–16 g/mL). Data were expressed as picomoles (pmol) of NO_2_^-^ and NO_3_^-^ per milligrams (mg) of proteins.

### 4.9. Measurement of Redox Status Activity

The total activity of superoxide dismutase (SOD), catalase (CAT), and glutathione peroxidase (GPx) were measured in K562 and Vero cells treated with 0.75 mg/mL of S.M. for 48 h. The tests were performed by commercially available ELISA (enzyme-linked immunosorbent assay) test kits, according to a previously reported protocol [55], and absorbances were measured spectrophotometrically (Glomax Multi detection System Spectrophotofluorimeter, Promega Corporation). Data were expressed in units (U) for milligrams (mg) of proteins.

### 4.10. Oxidative DNA Damage

Oxidative DNA damage was examined using a DNA damage (8-OHdG) ELISA kit (StressMarq Biosciences, Victoria, BC, Canada) [56], following the manufacturer’s instructions. The absorbance at 450 nm was measured by Glomax Multi Detection System Spectrophotofluorimeter (Promega Corporation, Milan, Italy). The content of 8-OHdG was calculated using a calibration curve and expressed in nanograms (ng) of 8-OHdG per mL of cell lysates.

### 4.11. Quantitative Real-Time PCR (RT-qPCR)

Total RNA was extracted using TRIsure (Bioline, Germany) according to manufacturer’s instructions, and its quantity was determined by NanoDrop. One microgram of total RNA was retro-transcribed using QuantiTect Reverse Transcription kit (QIAGEN, Milan, Italy), according to its protocol. qPCR analyses were performed using 20 ng cDNA per well in duplicate for each data point with Power SYBR green master mix (Thermo Fisher Scientific, Milan, Italy). The mRNA abundance of NOX2, p22-phox, Bax, Bcl-2, and GAPDH (as housekeeping gene) was analyzed using the oligos in Table 1.

The thermal cycling conditions were composed of 50 °C for 2 min followed by an initial denaturation step at 95 °C for 10 min, 40 cycles at 95 °C for 15 s, 60 °C for 1 min, a dis-sociation step at 95 °C for 15 s, and a final step at 60 °C for 15 s. The relative quantification in gene expression was determined using the 2^−ΔΔCt^ method [57] that allows to obtain the fold changes in gene expression normalized to an internal control gene.

### 4.12. Western Blot Analysis

For total protein extraction, treated and untreated K562 and Vero cells pellets were lysed on ice for 30 min in lysis buffer (1 mM EDTA, 150 mM NaCl, 1% NP-40, 50 mM TRIS-HCL (pH 7.5), supplemented with protease/phosphatase inhibitors). The protein concentration was determined by Bradford Assay, reading each sample at spectrophotometer (Bio-Rad Protein Assay Dye Reagent Concentrate Cat#5000006) at 595 nm, an absorbance proportional to the amount (concentration) of protein present in the sample. Mini-PROTEAN^®^ precast gel 4–12% (Bio-Rad, Milan, Italy) and Opti-Protein XL (Applied Biological Materials Inc., Richmond, BC, Canada) as molecular weight marker was used. Trans-Blot^®^ Turbo PVDF membrane (Bio-Rad, Milan, Italy) was used to transfer proteins. The membranes were probed with primary antibodies (dilution 1:1000): NOX2 (Rabbit polyclonal antibody, Cell Signaling, Leiden, The Netherlands) and p22-phox (Rabbit polyclonal antibody, Santa Cruz Biotechnology, Heidelberg, Germany); Bcl-2 (Rabbit polyclonal antibody, Cell Signaling, Leiden, The Netherlands); Bax (Rabbit polyclonal antibody, Cell Signaling, Leiden, The Netherlands); tubulin (Mouse monoclonal antibody, Santa Cruz Biotechnology, Heidelberg, Germany) as house-keeping protein; and secondary antibodies (dilution 1:2000). Blots were incubated with HRP (Horseradish Peroxidase) conjugated secondary antibodies (Santa Cruz Biotechnology, Heidelberg, Germany), according to the species of primary antibodies (dilution 1: 2000), and were developed using the ECL substrate (Bio-Rad, Milan, Italy). Signal intensity was quantified by the ChemiDoc™ Imaging System (Bio-Rad, Milan, Italy), with the Bio-Rad Quantity One^®^ software version 4.6.3. The results were expressed as arbitrary units.

### 4.13. Statistical Analysis

The GraphPad InStat Version 3.00 for Windows 95 (GraphPad Software, San Diego, CA, USA) was used for statistical analysis. Statistically significant differences were evaluated by Student’s *t*-test and one-way analysis of variance (ANOVA), followed by Tukey’s posttest. The experiments were performed at least in triplicates, and *p* <  0.05 was considered statistically significant.

## 5. Conclusions

This in vitro study, carried out on K562 cell line, showed that S.M. acts as an exogenous antioxidant and favors cancer cell death, highlighting its beneficial effect against CML.

Although the role of S.M. extract should be further investigated, these preliminary results elucidate its anti-apoptotic effect on K562 cells by modulating the antioxidant pathway, in particular NOX2 and its p22-phox subunit. 

Therefore, our pilot study offers fresh proof of the potential advantages of microalgal-derived compounds and their potential applications as an antioxidant adjuvant in the treatment of malignancies, particularly CML. 

## Figures and Tables

**Table 1:** Gene names, primer forward (F) and reverse (R) sequences, and amplicon size.

Gene	Accession NO.	Sequences of Primer Pairs (5′→3′)	Amplicon Size
NOX2	NM_000397.4	F: CTACAACATCTACCTCACTGGCTG	117
		R: GGCCGTCCATACAAAGTCTTT	
p22-phox	NM_000101.4	F: TGTGGCGGGCGTGTTTGTGT	241
		R: CAGTAGGTAGATGCCGCTCG	
Bax	NM_001291428.2	F: TCAGGATGCGTCCACCAAGAAG	103
		R: TGTGTCCACGGCGGCAATCATC	
Bcl-2	NM_000633.3	F: TCGCCCTGTGGATGACTGA	134
		R: CAGAGACAGCCAGGAGAAATCA	
GAPDH	NM_001256799.3	F: GAGTCAAGGGATTTGGTCGT	138
		R: GACAAGCTTCCCGTTCTCAG	

## Data Availability

The data sets used and/or analyzed during the current study are available from the corresponding author.

## Sample Availability

Not available.

## References

[1] Gangping H., Jing J., Hanming J., Yuanying Z., Mengdi W., Yuyu Q., Cundong F., Lijuan Y., Suyun B., Lingyun S. (2020). Acetylshikonin induces apoptosis of human leukemia cell line K562 by inducing S phase cell cycle arrest, modulating ROS accumulation, depleting Bcr-Abl and blocking NF-κB signaling. Biomed. Pharmacother..

[2] Ciarcia R., d’Angelo D., Pacilio C., Pagnini D., Galdiero M., Fiorito F., Damiano S., Mattioli E., Lucchetti C., Florio S. (2010). Dysregulated calcium homeostasis and oxidative stress in chronic myeloid leukemia (CML) cells. J. Cell. Physiol..

[3] Andretta E., Costa C., Longobardi C., Damiano S., Giordano A., Pagnini F., Montagnaro S., Quintiliani M., Lauritano C., Ciarcia R. (2021). Potential Approaches Versus Approved or Developing Chronic Myeloid Leukemia. Therapy. Front. Oncol..

[4] Simone C., Jane F.A. (2018). The argument for using imatinib in CML. Hematol. Am. Soc. Hematol. Educ. Prog..

[5] Richardson C., Yan S., Vestal C.G. (2015). Oxidative stress, bone marrow failure, and genome instability in hematopoietic stem cells. Int. J. Mol. Sci..

[6] Mohammadalipour A., Dumbali S.P., Wenzel P.L. (2020). Mitochondrial Transfer and Regulators of Mesenchymal Stromal Cell Function and Therapeutic Efficacy. Front. Cell Dev. Biol..

[7] Naughton R., Quiney C., Turner S.D., Cotter T.G. (2009). Bcr-Abl-mediated redox regulation of the PI3K/ AKT pathway. Leukemia.

[8] Rodrigues M.S., Reddy M.M., Sattler M. (2008). Cell cycle regulation by oncogenic tyrosine kinases in myeloid neoplasias: From molecular redox mechanisms to health implications. Antioxid. Redox Signal..

[9] Singh R.K., Tripathi A.K., Tripathi P., Singh S., Singh R., Ahmad R. (2009). Studies on biomarkers for oxidative stress in patients with chronic myeloid leukemia. Hematol. Oncol. Stem Cell Ther..

[10] Kaweme N.M., Zhou W., Changwe G.J., Zhou F. (2020). The significant role of redox system in myeloid leukemia: From pathogenesis to therapeutic applications. Biomark. Res..

[11] Frijhoff J., Winyard P., Zarkovic N., Davies S.S., Stocker R., Cheng D., Knight A.R., Taylor E.L., Oettrich J., Ruskovska T. (2015). Clinical Relevance of Biomarkers of Oxidative Stress. Antioxid. Redox Signal..

[12] Sillar J.R., Germon Z.P., De Iuliis G.N., Dun M.D. (2019). The role of reactive oxygen species in acute myeloid leukaemia. Int. J. Mol. Sci..

[13] Curta J.C., Rabello de Moraes A.C., Licínio M.A., Costa A., Santos-Silva M.C. (2012). Effect of nitric oxide on the daunorubicin efflux mechanism in K562 cells. Cell Biol. Int..

[14] Bedard K., Krause K.H. (2007). The NOX family of ROS-generating NADPH oxidases: Physiology and pathophysiology. Physiol. Rev..

[15] Sardina J.L., López-Ruano G., Sánchez-Abarca L.I., Pérez-Simón J.A., Gaztelumendi A., Trigueros C., Llanillo M., Sánchez-Yagüe J., Hernández-Hernández A. (2010). p22phox-dependent NADPH oxidase activity is required for megakaryocytic differentiation. Cell Death Differ..

[16] Saide A., Damiano S., Ciarcia R., Lauritano C. (2021). Promising Activities of Marine Natural Products against Hematopoietic Malignancies. Biomedicines.

[17] Schwartsmann G., Brondani da Rocha A., Berlinck R.G., Jimeno J. (2001). Marine organisms as a source of new anticancer agents. Lancet Oncol..

[18] Bourbon E., Salles G. (2020). Polatuzumab vedotin: An investigational anti-CD79b antibody drug conjugate for the treatment of diffuse large B-cell lymphoma. Expert Opin. Investig. Drugs.

[19] Ketchum E.B., Clarke A., Clemmons A.B. (2022). Belantamab Mafodotin-blmf: A Novel Antibody-Drug Conjugate for Treatment of Patients With Relapsed/Refractory Multiple Myeloma. J. Adv. Pract. Oncol..

[20] Miralto A., Barone G., Romano G., Poulet S.A., Ianora A., Russo G.L. (1999). The insidious effect of diatoms on copepod reproduction. Nature.

[21] Sarno D., Kooistra W.H.C.F., Medlin L.K., Percopo I., Zingone A. (2005). Diversity in the genus Skeletonema (Bacillariophyceae). ii. An assessment of the taxonomy of *S. costatum*-like species with the description of four new species. J. Phycol..

[22] Ingebrigtsen R.A., Hansen E., Andersen J.H., Eilertsen H.E. (2016). Light and temperature effects on bioactivity in diatoms. J. Appl. Phycol..

[23] Lauritano C., Andersen J.H., Hansen E., Albrigtsen M., Escalera L., Esposito F., Helland K., Hanssen K. (2016). Romano, G.; Ianora, A. Bioactivity Screening of Microalgae for Antioxidant, Anti-Inflammatory, Anticancer, Anti-Diabetes, and Antibacterial Activities. Front. Mar. Sci..

[24] Lauritano C., Carotenuto Y., Vitiello V., Buttino I., Romano G., Hwang J.S., Ianora A. (2015). Effects of the oxylipin-producing diatom *Skeletonema marinoi* on gene expression levels in the calanoid copepod *Calanus sinicus*. Mar. Genom..

[25] Fontana A., D’Ippolito G., Cutignano A., Miralto A., Ianora A., Romano G. (2007). Chemistry of oxylipin pathways in marine diatoms. Pure Appl. Chem..

[26] Miceli M., Cutignano A., Conte M., Ummarino R., Romanelli A., Ruvo M., Leone M., Mercurio F.A., Doti N., Manzo E. (2019). Monoacylglycerides from the Diatom *Skeletonema marinoi* Induce Selective Cell Death in Cancer Cells. Mar. Drugs.

[27] Saide A., Martínez K.A., Ianora A., Lauritano C. (2021). Unlocking the Health Potential of Microalgae as Sustainable Sources of Bioactive Compounds. Int. J. Mol. Sci..

[28] Smerilli A., Orefice I., Corato F., Ruban A., Brunet C. (2017). Photoprotective and antioxidant responses to light spectrum and intensity variations on a coastal diatom. Environ. Microbiol..

[29] Brillatz T., Lauritano C., Jacmin M., Khamma S., Marcourt L., Righi D., Romano G., Esposito F., Ianora A., Queiroz E.F. (2018). Zebrafish-based identification of the antiseizure nucleoside inosine from the marine diatom *Skeletonema marinoi*. PLoS ONE.

[30] Lauritano C., Romano G., Roncalli V., Amoresano A., Fontanarosa C., Bastianini M., Braga F., Carotenuto Y., Ianora A. (2016). New oxylipins produced at the end of a diatom bloom and their effects on copepod reproductive success and gene expression levels. Harmful Algae.

[31] d’Ippolito G., Cutignano A., Briante R., Febbraio F., Cimino G., Fontana A. (2005). New C16 fatty-acid-based oxylipin pathway in the marine diatom *Thalassiosira rotula*. Org. Biomol. Chem..

[32] Lauritano C., Carotenuto Y., Miralto A., Procaccini G., Ianora A. (2012). Copepod population-specific response to a toxic diatom diet. PLoS ONE.

[33] Hockenbery D.M., Oltval Z.N., Yin X.M., Milliman C.L., Korsmeyer S.J. (1993). Bcl-2 functions in an antioxidant pathway to prevent apoptosis. Cell.

[34] Chipuk J.E., Bouchier-Hayes L., Green D.R. (2006). Mitochondrial outer membrane permeabilization during apoptosis: The innocent bystander scenario. Cell Death Differ..

[35] Korsmeyer S.J., Shutter J.R., Veis D.J., Merry D.E., Oltvai Z.N. (1993). Bcl-2/Bax: A rheostat that regulates an anti-oxidant pathway and cell death. Semin. Cancer Biol..

[36] Veis D.J., Sorenson C.M., Shutter J.R., Korsmeyer S.J. (1993). Bcl-2-deficient mice demonstrate fulminant lymphoid apoptosis, polycystic kidneys, and hypopigmented hair. Cell.

[37] Hanusova V., Skalova L., Kralova V., Matouskova P. (2015). Potential anti-cancer drugs commonly used for other indications. Curr. Cancer Drug Targets.

[38] Riyasat A., Mirza Z., Ghulam M.D.A., Mohammad A.K., Shakeel A.A., Ghazi A.D., Adel M.A., Adeel G.C., Ishfaq A.S. (2012). New anticancer agents: Recent developments in tumor therapy. Anticancer Res..

[39] Ozkan G., Ulusoy S., Orem A., Alkanat M., Mungan S., Yulug E., Yucesan F.B. (2013). How Does Colistin-Induced Nephropathy Develop and Can It Be Treated?. Antimicrob. Agents Chemother..

[40] Korhonen R., Lahti A., Kankaanranta H., Moilanen E. (2005). Nitric Oxide Production and Signaling in Inflammation. Curr. Drug Targets Inflamm. Allergy.

[41] Longobardi C., Damiano S., Andretta E., Prisco F., Russo V., Pagnini F., Florio S., Ciarcia R. (2021). Curcumin Modulates Nitrosative Stress, Inflammation, and DNA Damage and Protects against Ochratoxin A-Induced Hepatotoxicity and Nephrotoxicity in Rats. Antioxidants.

[42] Valko M., Leibfritz D., Moncol J., Cronin M.T., Mazur M., Telser J. (2007). Free radicals and antioxidants in normal physiological functions and human disease. Int. J. Biochem. Cell Biol..

[43] Clerkin J.S., Naughton R., Quiney C., Cotter T.G. (2008). Mechanisms of ROS modulated cell survival during carcinogenesis. Cancer Lett..

[44] Irwin M.E., Rivera-Del Valle N., Chandra J. (2013). Redox control of leukemia: From molecular mechanisms to therapeutic opportunities. Antioxid. Redox Signal..

[45] Rizwan A., Tripathi A., Tripathi P., Singh R., Singh S., Singh R. (2008). Oxidative stress and antioxidant status in patients with chronic myeloid leukemia. Indian J. Clin. Biochem..

[46] Slupianek A., Poplawski T., Jozwiakowski S.K., Cramer K., Pytel D., Stoczynska E., Nowicki M.O., Blasiak J., Skorski T. (2011). BCR/ABL stimulates WRN to promote survival and genomic instability. Cancer Res..

[47] Singh M.M., Irwin M.E., Gao Y., Ban K., Shi P., Arlinghaus R.B., Amin H.M., Chandra J. (2012). Inhibition of the NADPH oxidase regulates heme oxygenase 1 expression in chronic myeloid leukemia. Cancer.

[48] Torres-Tiji Y., Fields F.J., Mayfield S.P. (2020). Microalgae as a future food source. Biotechnology.

[49] Fradique M., Batista A.P., Nunes C.M., Gouveia L., Bandarra N.M., Raymundo A. (2013). *Isochrysis galbana* and *Diacronema vlkianum* biomass incorporation in pasta products as PUFA’s source. LWT—Food Sci. Technol..

[50] Gouveia L., Coutinho C., Mendonça E., Batista A.P., Sousa I., Bandarra N.M., Raymundo A. (2008). Functional biscuits with PUFA-ω3 from Isochrysis galbana. J. Sci. Food Agric..

[51] Martínez K.A., Saide A., Crespo G., Martín J., Romano G., Reyes F., Lauritano C., Ianora A. (2022). Promising Antiproliferative Compound From the Green Microalga *Dunaliella tertiolecta* Against Human Cancer Cells. Front. Mar. Sci..

[52] Montagnaro S., Damiano S., Ciarcia R., Puzio M.V., Ferrara G., Iovane V., Forte I.M., Giordano A., Pagnini U. (2019). Caprine herpesvirus 1 (CpHV-1) as a potential candidate for oncolytic virotherapy. Cancer Biol. Ther..

[53] Ohkawa H., Ohishi N., Yagi K. (1979). Assay for lipid peroxide in animal tissues by thiobarbituric acid reaction. Anal. Biochem..

[54] Florio S., Ciarcia R., Crispino L., Pagnini U., Ruocco A., Kumar C., D’Andrilli G., Russo F. (2003). Hydrocortisone has a protective effect on CyclosporinA-induced cardiotoxicity. J. Cell. Physiol..

[55] Tsai M.C., Huang T.L. (2016). Increased activities of both superoxide dismutase and catalase were indicators of acute depressive episodes in patients with major depressive disorder. Psychiatry Res..

[56] Onuma S., Manabe A., Yoshino Y., Matsunaga T., Asai T., Ikari A. (2021). Upregulation of Chemoresistance by Mg^2+^ Deficiency through Elevation of ATP Binding Cassette Subfamily B Member 1 Expression in Human Lung Adenocarcinoma A549 Cells. Cells.

[57] Livak K.J., Schmittgen T.D. (2001). Analysis of relative gene expression data using real-time quantitative PCR and the 2(-Delta Delta C(T)) Method. Methods.

